# Variations in Anthocyanin Profiles and Antioxidant Activity of 12 Genotypes of Mulberry (*Morus* spp.) Fruits and Their Changes during Processing

**DOI:** 10.3390/antiox9030242

**Published:** 2020-03-17

**Authors:** Inhwan Kim, Jihyun Lee

**Affiliations:** Department of Food Science and Technology, Chung-Ang University, Anseong 17546, Korea; hgodos@daum.net

**Keywords:** genotype, cyanidin, cyanidin glycoside, malvidin, petunidin, UHPLC-(ESI)-qTOF, syrup, citric acid, high resolution mass spectroscopy, DPPH

## Abstract

Mulberry fruits are known as rich sources of anthocyanins and are consumed in syrup form after the addition of sugar and acid; however, there is little information on the anthocyanin composition and antioxidant activity of mulberries of different cultivars and their changes during processing. To address this, the antioxidant activity and anthocyanin composition of 12 cultivar mulberry fruit cultivars were investigated by high-performance liquid chromatography and ultra-high-performance liquid chromatography coupled with electrospray ionization/quadrupole time-of-flight. Additionally, different quantities of citric acid were used to evaluate antioxidant activities and anthocyanin composition of mulberry syrup. Sixteen anthocyanins were identified in mulberry fruits using accurate mass spectrometry. Several anthocyanins were tentatively identified for the first time in mulberry fruits and include: malvidin hexoside, cyanidin malonyl hexose hexoside, cyanidin pentoside, cyanidin malonyl hexoside, petunidin deoxyhexose hexoside, and cyanidin deoxyhexoside. The major anthocyanin in mulberries was cyanidin-3-*O*-glucoside, followed by cyanidin-3-*O*-rutinoside. *Morus Alba* L. Iksu showed the highest cyanidin-3-*O*-glucoside content (8.65 mg/g dry weight) among 12 mulberry fruit cultivars. As citric acid levels increased, mulberry syrup showed significantly higher antioxidant activity (*p* < 0.05).

## 1. Introduction

Mulberry leaves (*Morus* species) are cultivated as feed for silkworms in China and India. A recent clinical study showed that mulberry leaf extracts may lower the blood sugar levels of patients with type 2 diabetes [1]. An in vitro study showed that mulberry fruit extracts have effects on antidiabetic and antioxidant effects [2]. Furthermore, mulberry fruits have been consumed to lower fever and protect against liver disease [3]. With wellness trends spreading worldwide, mulberry fruit consumption has increased in Asia due to its high anthocyanin contents. In Korea, mulberry fruit yields increased twofold from 2008 to 2011 [4].

Major sources of anthocyanin, a natural pigment, include berries [3,5]. Various studies have shown that high-anthocyanin diets are highly correlated with anti-inflammatory, anti-cancer, and antioxidant effects [6,7]. The anthocyanin composition depends on the berry type; furthermore, anthocyanins are present in glycosidic forms, which vary with berry type. For example, bilberry fruits contain cyanidin-3-*O*-galactoside, cyanidin-3-*O*-glucoside, and cyanidin-3-*O*-arabinoside [5]. Raspberries contain cyanidin glycosides, which vary from monomeric to trimeric forms [8]. Moreover, the main anthocyanins in strawberries and blackberries are pelargonidin-3-*O*-glucoside and cyanidin-3-*O*-glucoside [5]. Our recent study showed that mulberry fruits (*Morus alba* L.) contain cyanidin-3-*O*-glucoside and cyanidin-3-*O*-rutinoside as the primary anthocyanins [9]. Anthocyanins are 23 times more abundant in mulberry fruits than in grapes [10]. In addition to the berry type, anthocyanin composition may vary with cultivars, environmental conditions (e.g., temperature and UV exposure), agricultural practices (e.g., organic vs. conventional cropping), processing, and storage. You et al. (2011) reported that the ‘Climax’ blueberry cultivar contains 1.5-folds higher anthocyanin contents than ‘Powderblue’ blueberry cultivar and the anthocyanin composition was different in the two cultivars; however, there was no significant difference in the anthocyanin composition of the organically and conventionally grown blueberry samples [11]. Recent studies showed that high-pressure processing treatment enhances anthocyanin stability in mulberry juice and anthoycyanin contents decrease during storage [12,13]. The chemical composition of mulberry fruits including sugar, organic acid, and fatty acid contents was previously reported [14,15]. However, there are few studies on the differences in anthocyanin composition according to mulberry fruit cultivars and processing.

Mulberry fruits are stored frozen or processed as syrup after harvest due to their short shelf life [16]. Anthocyanins in mulberry fruits have been analyzed by high-performance liquid chromatography (HPLC) [17,18]. One study analyzed the anthocyanin composition of mulberries using triple quadrupole mass spectrometry (LC-QqQ) [19]. However, LC-QqQ only provides the unit mass of target pseudomolecular ions and is thus unsuitable for the identification of unknown pseudomolecular ions. However, accurate mass analyzers, such as quadrupole time-of-flight (qTOF) mass spectrometers, offer accurate results of the masses of precursor and product ions. Particularly, obtaining an accurate fragmental ion mass may aid in interpreting structural information [20].

In this study, the antioxidant activity, total phenolic content (TPC), and total flavonoid content (TFC) of 12 mulberry fruit cultivars were investigated. Additionally, the anthocyanin composition was investigated with HPLC and ultra-high-performance liquid chromatography coupled with electrospray ionization/quadrupole time-of-flight (UHPLC-(ESI)-qTOF). Furthermore, we determined the influence of citric acid addition during mulberry syrup production on antioxidant activity and anthocyanin contents.

## 2. Materials and Methods

### 2.1. Mulberry Fruit Samples

Twelve mulberry fruit cultivars were purchased from Yangpyeong Yangjam Youngnong Co., Ltd. (a sericulture corporation; Yangpeyong, Korea; 37°26′40.6″N, 127°46′04.0″E). Plants were watered every 2–3 weeks or as needed. All fruit cultivars were cultivated in the same type of soil as well as under the same environmental conditions. Mulberry fruits were harvested at commercial maturity in mid-June 2016. About 3–5 kg of fresh mulberry fruits per cultivar were used to produce a composite sample. For sugar and color analysis, mulberry juice was freshly prepared. For other analyses, the composite samples were freeze-dried and stored at −80 °C until analysis. Then, the freeze-dried sample was ground and sieved to obtain a uniform size.

### 2.2. Chemicals and Reagents

Anthocyanin compound standards (cyanidin-3-*O*-glucoside, cyanidin-3-*O*-rutinoside, and pelargonidin-3-*O*-glucoside) were purchased from Extrasynthese (Lyon Nord, France). Gallic acid, quercetin, trolox, HPLC-grade trifluoroacetic acid (TFA) and hydrochloric acid were purchased from Sigma-Aldrich (St. Louis, MO, USA). HPLC-grade methanol and acetonitrile were obtained from Burdick & Jackson (Muskegon, MI, USA). Nanopure water was obtained from a water purification system (Milli-Q Direct 8, Merck Millipore, Billerica, MA, USA).

### 2.3. Determination of Soluble Solid Contents and Color of Mulberry Fruit Juice

Soluble solid contents and colors of mulberry fruit juices were investigated using a refractometer (PAL-1, ATAGO, Tokyo, Japan) and HunterLab model UltraScan PRO colorimeter (Hunterlab Reston, VA, USA), respectively. The hunter color values, L, a, and b, represent whiteness (0: black, 100: white), redness (positive a values indicate redness; negative a values indicate greenness), and yellowness (positive b values describe yellowness; negative b values describe blueness), respectively. Calibration was conducted using a standard white tile (L = 93.48, a = −0.93, b = 0.41).

### 2.4. Analysis of TPC, TFC, and Antioxidant Activities

First, 500 mg of lyophilized mulberry powder was mixed with 10 mL of 60% aqueous methanol. The mixture was sonicated for 15 min after homogenization and then centrifuged at 10,621× *g* for 15 min. The supernatant was filtered through a 0.45 µm polytetrafluoroethylene (PTFE) syringe filter.

TPC values of the mulberry fruit extracts were measured by a modified method of Ceymann et al. [21]. In brief, 40 µL of the extracts was mixed with 50 µL of 1 N Folin-Ciocalteu reagent and 160 µL of 2% aqueous sodium carbonate solution and allowed to react for 30 min. The absorbance was measured at 700 nm using a spectrophotometer (Multiskan GO, Thermo Scientific, Waltham, MA, USA). TPC was expressed as mg gallic acid equivalent (GAE)/g dry weight (DW).

TFC values of the mulberry fruit extracts were investigated by the method of Moreno et al. with slight modification [22,23,24]. Extracts (25 μL) were reacted with 225 µL of a reagent mixture comprising 10% aqueous aluminum nitrate, 1 M potassium acetate, and methanol for 40 min. The absorbance was measured at 415 nm. TFC was expressed as mg quercetin equivalent/g DW. Aluminum nitrate method is specific to flavonols and flavones [23,25,26]. Thus, the flavonoids determined in the TFC in this study would be mainly flavonols and flavones.

Antioxidant activities of the mulberry extracts were evaluated by 2,2-diphenyl-1-picrylhydrazyl (DPPH) radical scavenging activity and ferric ion reducing antioxidant power (FRAP) assay. DPPH radical scavenging activity was determined according to a previously described method with some modification [27]. Extracts (150 μL) were reacted with 200 µL of 0.15 mM DPPH solution dissolved in ethanol for 15 min. The absorbance was measured at 517 nm. FRAP assay was conducted by a modified method of Thaipong et al. [28]. The FRAP reagent comprised 300 mM acetate buffer, 10 mM 2,4,6-tris(2-pyridyl)-s-triazine (TPTZ) dissolved in 40 mM aqueous hydrochloric acid solution, and 20 mM iron (III) chloride, and 240 µL of the FRAP reagent was reacted with mulberry extracts for 30 min. The absorbance was measured at 593 nm. The DPPH and FRAP assay results were expressed as mg trolox equivalent/g DW.

### 2.5. Analysis of Anthocyanin Composition in Mulberry Fruits Using HPLC

The anthocyanin composition of mulberry fruits was analyzed by our previously reported method [9]. First, 0.3 g of lyophilized mulberry powder was mixed with 30 mL of 0.1% HCl in methanol. The extract was sonicated for 30 min and centrifuged at 10,621× *g* for 15 min. The extraction was repeated. The supernatant was filtered using a 0.22 µm polyvinylidene fluoride (PVDF) syringe filter for analysis.

The anthocyanin composition in mulberry extracts was determined by HPLC using the Agilent 1260 Infinity II LC system (Agilent Technologies, Santa Clara, CA, USA) with a photo diode array detector. Mobile phases were 1% TFA in water (A) and 1% TFA in acetonitrile (B). The mobile phase gradient was as follows: 0–6.5 min, 10%–12% (B); 6.5–10.5 min, 12%–13% (B); 10.5–33 min, 13%–17% (B); 33–60 min, 17%–65% (B); and 60–70 min, 65%–95% (B). The injection volume was 20 µL and the flow rate was 1 mL/min. Anthocyanin compounds (i.e., cyanidin-3-*O*-glucoside, cyanidin-3-*O*-rutinoside, and pelargonidin-3-*O*-glucoside) in mulberry extracts were separated by a Zorbax Eclipse XDB-C_18_ column (4.6 × 250 mm, 5 µm, Agilent Technologies) at 40 °C. The wavelength was monitored at 520 nm. Anthocyanins were quantified using the authentic standards of cyanidin-3-*O*-glucoside, cyanidin-3-*O*-rutinoside, and pelargonidin-3-*O*-glucoside.

### 2.6. Identification of Anthocyanins by UHPLC-(ESI)-qTOF

The anthocyanin composition in mulberry fruit extracts was further analyzed by an Agilent 1290 Infinity UHPLC system coupled to a 6530 accurate mass qTOF mass spectrometer with electrospray ionization (UHPLC-(ESI)-qTOF MS/MS) (Agilent Technologies). The anthocyanin compounds of the extracts were separated by a Poroshell C18 column (2.1 × 100, 2.7 μm, Agilent Technologies) at 30 °C. The mobile phases consisted of a gradient of 1% formic acid in water (A) and 1% formic acid in acetonitrile (B) in ESI positive mode. TFA was replaced with formic acid to prevent ion suppression caused by TFA. The injection volume was 5 μL. The other LC-(ESI)-MS methods followed our previously reported method [29]. The drying gas flow rate, temperature, nebulizer gas pressure, fragment voltage, and skimmer voltage were 8.0 L/min, 225 °C, 45 psi, 125 V, and 65 V, respectively. The capillary voltage was 3.5 kV.

An anthocyanin database that contained possible anthocyanin compounds from previous literature was developed. The database provided theoretical masses of possible anthocyanins. Anthocyanins were identified by comparisons between the theoretical and observed *m*/*z*. Isotope abundance and spacing were also compared using Masshunter Qualitative Analysis (Agilent Technologies). Then, the identified anthocyanin peaks were further investigated for fragmentation patterns using MS/MS mode using 20 eV collision energy. For MS/MS analysis, the scan rate was 3 spectra/sec and 2 maximum precursors/cycle. Mass-to-charge ratios (*m*/*z*) of 100–1000 were monitored. If available, MS1 (retention time, mass, and isotope patterns) and MS2 data (fragmentation patterns) of the mulberry extracts were compared with authentic standards. The extracted ion peak area at its retention time was used for relative quantification of each anthocyanin compound in the mulberry fruit extract by qTOF in MS1 mode.

### 2.7. Determination of Anthocyanin Composition in Mulberry Syrup

Mulberry syrup was produced by adding sugar and citric acid to mulberry fruits purchased from a local market in Anseong, Korea. In Korea, mulberry syrup is often made with sugar and citric acid by allowing the mixture to stand for 2 weeks at ambient temperature (25 °C) for consumption. Thus, sugar was mixed with mulberry fruits in a ratio of 1:1 (w/w). Then, citric acid was added at 0% (control), 0.2%, or 0.3% (w/w) of mulberry fruits. After storing for 2 weeks at 25 °C, mulberry syrup was separated from solid residues. Antioxidant activities (DPPH assay), TPC, TFC, and the anthocyanin composition were determined in the syrup and solid residues as described in 2.4 and 2.5. Because the solid residues are often consumed with syrup, they were also analyzed.

### 2.8. Statistical Analysis

Statistical analyses were performed using IBM SPSS Statistics 23 (v. 23.0, SPSS, Inc., Chicago, IL, USA). Significant differences among mulberry cultivars and processing conditions of mulberry syrup were determined using one-way ANOVA followed by a post-hoc test, Duncan’s multiple range test, at *p* < 0.05. Principal component analysis and a heatmap for the peak area of anthocyanins identified by UHPLC-(ESI)-qTOF were performed using XLSTAT (ver. 2017.03, Microsoft Excel Add-in Software, New York, NY, USA).

## 3. Results and Discussion

Table 1 shows the basic information of the mulberry samples, including the breeding year, genetic information, species, and fruit weight of 12 mulberry fruit cultivars.

### 3.1. Soluble Solid Contents and Color of Mulberry Fruit Juice

Table 1 shows the soluble solid content in 12 mulberry cultivars, which ranged from 10.5 to 18.3 °Brix; in a previous report, these values were 5.66 to 15.62 °Brix [30]. Our results were similar to the previously reported values. Hunter color values are shown in Table S1. The ranges of lightness (L), redness (a), and yellowness (b) were 41.45–80.99, 29.75–72.69, and −1.16–68.82, respectively. Daeja mulberry fruits showed significantly higher L and lower a and b values than that showed by other cultivars (*p* < 0.05).

### 3.2. Antioxidant Activities, TPC, and TFC in Mulberry Fruits

Table 2 shows the antioxidant activities, TPC, and TFC of 12 mulberry fruit cultivars. DPPH radical scavenging activity of mulberry fruits ranged from 5.85 to 40.73 mg trolox equivalent/g DW. DPPH radical scavenging activity of mulberries cultivated at China ranged from 0.44 to 21.03 mg trolox equivalent/g DW with a 90% estimated moisture content [31]. The FRAP of 12 mulberry cultivars ranged from 1.33 to 82.87 mg trolox equivalent/g DW. Shimgang and Iksu cultivars showed significantly higher DPPH and FRAP results than other cultivars (*p* < 0.05).

The TPC of mulberries ranged from 5.68 to 40.46 mg GAE g DW. In accordance with our result, the TPC of mulberry fruits harvested in Korea was reported to be as high as 9.59 to 25.7 mg GAE/g DW with a 90% estimated moisture content [3]. Iksu and Shimgang had significantly higher TPC (38.31–40.46 mg GAE/g DW) than the reported values (*p* < 0.05). Total phenolic content of mulberries cultivated in China ranged from 1.99 to 23.30 mg GAE/g DW with a 90% estimated moisture content [31]. The twelve cultivars showed TFC ranging from 0.65 to 3.70 mg quercetin equivalent/g DW; these contents were higher in Shimgang and Iksu (3.49–3.70 mg quercetin equivalent/g DW) than in other cultivars (*p* < 0.05). The higher TPC and TFC values of Iksu and Shimgang mulberries may explain their higher antioxidant activities.

### 3.3. Anthocyanin Composition of Mulberries Determined by HPLC

Table 3 shows the anthocyanin contents in 12 mulberry cultivars. Cyanidin-3-*O*-glucoside, cyanidin-3-*O*-rutinoside, and pelargonidin-3-*O*-glucoside were measured in mulberry fruits by comparing the retention time and UV/vis spectra with those of authentic standards. The total anthocyanin contents (sum of individual anthocyanin levels) varied by cultivar and ranged from 0.51 (Daeja) to 28.61 (Iksu) mg/g DW. Cyanidin-3-*O*-glucoside was the major anthocyanin compound (ca 67% of total) in mulberries, which agreed with our previous results regarding anthocyanins in mulberry fruits [5]. Cyanidin-3-*O*-glucoside contents in mulberry fruits ranged from 0.33 (Daeja) to 19.51 mg/g DW (Iksu). Similar to our result, mulberries (cultivar information unknown) cultivated in Korea were reported to contain as much as 4.11 to 52.88 mg/g DW cyanidin-3-*O*-glucoside [32]. However, in our study, Daeja (0.33 mg/g DW) and Hasusang (0.62 mg/g DW) showed lower anthocyanin contents than reported values. Daeja fruits showed lower a values (redness) than others, which might be explained by their lower anthocyanin content (Table S1). Mulberry fruits (*Morus nigra* L.) grown in Italy exhibited cyanidin-3-*O*-glucoside levels of 17.9 mg/g DW [33]. Mulberry fruits harvested in China (*Morus alba* L., *Morus atropurpurea* Roxb., and *Morus multicaulis* Perr.) contained up to 33.5 mg/g DW (minimum level undetermined) of cyanidin-3-*O*-glucoside after correcting fresh weight (FW) to DW based on a 90% estimated moisture content [34].

Cyanidin-3-*O*-rutinoside contents ranged from 0.18 (Daeja) to 8.65 mg/g DW (Shimgang); this agreed with a previous study, which reported cyanidin-3-*O*-rutinoside contents between 1.12 to 22.92 mg/g DW [32]. Mulberry fruits (*Morus nigra* L.) grown in ltaly had lower cyanidin-3-*O*-rutinoside levels (7.5 mg/g DW) than those of Iksu and Shimgang (*Morus alba* L. and *Morus Microphylla* Buckl.) mulberry fruits (8.58–8.65 mg/g DW) [33]. Mulberry fruits cultivated in China showed up to 15.0 mg/g DW cyanidin-3-*O*-rutinoside after correcting FW to DW based on a 90% estimated moisture content [34].

Pelargonidin-3-*O*-glucoside ranged from undetermined levels (Daeja) to 0.51 mg/g DW (Iksu). Daeja contained the lowest anthocyanin content among the 12 mulberry fruit cultivars. It was reported that mulberries contained as much as 0.17 to 2.7 mg/g DW pelargonidin-3-*O*-glucoside after correcting FW values to DW based on a 90% estimated moisture content [35]; our result was within this range. Mulberry fruits (*Morus nigra* L.) grown in ltaly had higher pelargonidin-3-*O*-glucoside levels (1.2 mg/g DW) than the fruits investigated in this study (n.d.–0.51 mg/g DW) [33].

### 3.4. Determination of Anthocyanins in Mulberry Fruits Using UHPLC-(ESI)-qTOF

Table 4 presents anthocyanins identified in mulberry fruits using UHPLC-(ESI)-qTOF. The extracted ion chromatogram of Shimgang is shown in Figure S1. Sixteen anthocyanin compounds were identified. To the best of our knowledge, malvidin hexoside, cyanidin malonyl hexose hexoside, cyanidin pentoside, cyanidin malonyl hexoside, petunidindeoxyhexose hexoside, and cyanidin deoxyhexoside were tentatively identified for the first time in mulberry fruits. Cyanidin hexose deoxyhexose hexoside (peak 1) produced characteristic fragment ions at *m*/*z* 595.1657 after the loss of 162.0528 *m*/*z* corresponding to a hexose moiety, *m*/*z* 449.1078 after the loss of hexose and deoxyhexose moieties, and *m*/*z* 287.0550 after the loss of two hexose and deoxyhexose moieties. The *m*/*z* at 287.0550 indicates cyanidin aglycone. Cyanidin dihexoside (peak 2) produced fragment ions at *m*/*z* 449.1078 and 287.0550 after the loss of one and two hexose moieties, respectively. Cyanidin hexose deoxyhexose hexoside and cyanidin dihexoside (peaks 1 and 2) may be cyanidin glucosyl rutinoside and cyanidin diglucoside, respectively. Delphinidin dihexoside (peak 3) produced fragment ions at *m*/*z* 465.1028 and *m*/*z* 303.0499 after the loss of one and two hexose moieties, respectively; the latter indicates delphinidin aglycone.

Peaks 4, 5, and 6 were identified as cyanidin-3-*O*-glucoside, cyanidin-3-*O*-rutinoside, and pelargonidin-3-*O*-glucoside, respectively. The three identified anthocyanins were confirmed by comparing their retention times and MS/MS fragmentation patterns with those of authentic standards. Cyanidin-3-*O*-glucoside produced a characteristic fragment ion at *m*/*z* 287.0550 after the loss of a hexose moiety. Cyanidin-3-*O*-rutinoside produced fragment ions at *m*/*z* 449.1078 after the loss of a rhamnose moiety and *m*/*z* 287.0550 after the loss of rhamnose and glucose moieties.

Malvidin hexoside (peak 7) produced a fragment ion at *m*/*z* 331.0812 after the loss of a hexose moiety, indicating malvidin aglycone. Pelargonidin deoxyhexose hexoside (peak 8) produced fragment ions at *m*/*z* 433.1129 after the loss of a deoxyhexose moiety and *m*/*z* 271.0601 after the loss of deoxyhexose and hexose moieties; the latter indicates pelargonidin aglycone. It was reported that mulberry fruits contain pelargonidin-3-*O*-rutinoside [33]. Thus, it seems that pelargonidin deoxyhexose hexoside may be pelargonidin-3-*O*-rutinoside. Peonidin hexoside (peak 9) produced a fragment ion at *m*/*z* 301.0707 resulting from the loss of a hexose moiety, indicating peonidin aglycone.

Cyanidin malonyl hexose hexoside (peak 10) produced fragment ions at *m*/*z* 449.1078 resulting from the loss of hexose and malonic acid moieties and at *m*/*z* 287.0550 due to the loss of one malonic acid and two hexose moieties. Cyanidin pentoside (peak 11) produced a fragment ion at *m*/*z* 287.0550 resulting from the loss of a pentose moiety. Peonidin deoxyhexose hexoside (peak 12) exhibited fragment ions at *m*/*z* 463.1235 after the loss of a deoxyhexose moiety and *m*/*z* 301.0707 after the loss of deoxyhexose and hexose moieties. Cyanidin malonyl hexoside (peak 13) produced a fragment ion at *m*/*z* 287.0550 resulting from the loss of hexose and malonic acid moieties. Petunidin deoxyhexose hexoside (peak 14) produced a fragment ion at *m*/*z* 479.1184 after the loss of a deoxyhexose moiety and *m*/*z* 317.0656 after the loss of deoxyhexose and hexose moieties. Cyanidin deoxyhexoside (peak 15) produced a fragment ion at *m*/*z* 287.0550, losing a hexose moiety. Delphinidin deoxyhexose hexoside (peak 16) showed fragment ions at *m*/*z* 465.1028 and 303.0499 after the loss of deoxyhexose or deoxyhexose and hexose moieties, respectively. Delphinidin deoxyhexose hexoside may be delphinidin-3-*O*-rutinoside, which is reported to be contained in mulberry fruits [36].

**Figure 1:**
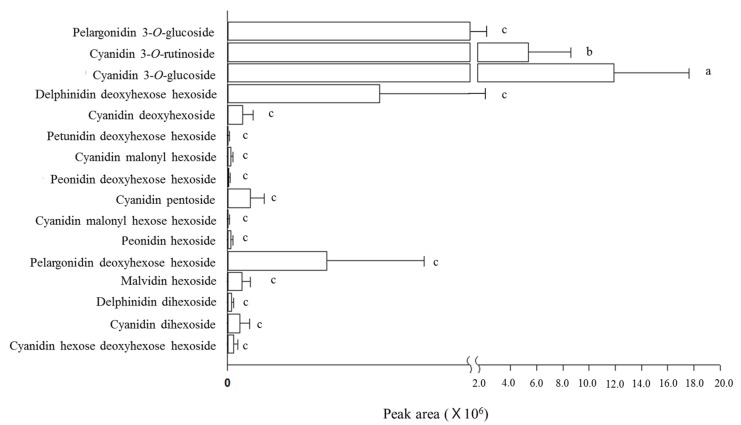
Averages of anthocyanin peak areas determined by UHPLC-(ESI)-qTOF in mulberry fruits of 12 cultivars.

The average extracted ion chromatogram (EIC) peak areas of anthocyanin peaks in 12 mulberry cultivar extracts are shown in Figure 1 and Table S2. Based on the peak areas, cyanidin-3-*O*-glucoside, cyanidin-3-*O*-rutinoside, and pelargonidin-3-*O*-glucoside were the predominant anthocyanin compounds in mulberry fruits. Averages of cyanidin-3-*O*-glucoside and cyanidin-3-*O*-rutinoside peak areas of 12 mulberry cultivars were significantly higher than those of other anthocyanins (*p* < 0.05). Because the peak area difference between the cultivars was large, those of other anthocyanins did not show significant differences (*p* > 0.05). As shown in the HPLC data, the EIC areas of cyanidin-3-*O*-glucoside, cyanidin-3-*O*-rutinoside and pelargonidin-3-*O*-glucoside were higher in Iksu and Shimgang mulberries than in other mulberry cultivars. Iksu and Shimgang contained all identified anthocyanin compounds shown in Table 4. When the anthocyanin peak areas were summed, a 22.6-fold difference was found among mulberry cultivars (14,002,119 in Daeja mulberries compared to 316,438,755 in Shimgang mulberries).

### 3.5. Principal Component Analysis and Heatmap Based on Mulberry Anthocyanins Determined by UHPLC-(ESI)-qTOF

**Figure 2:**
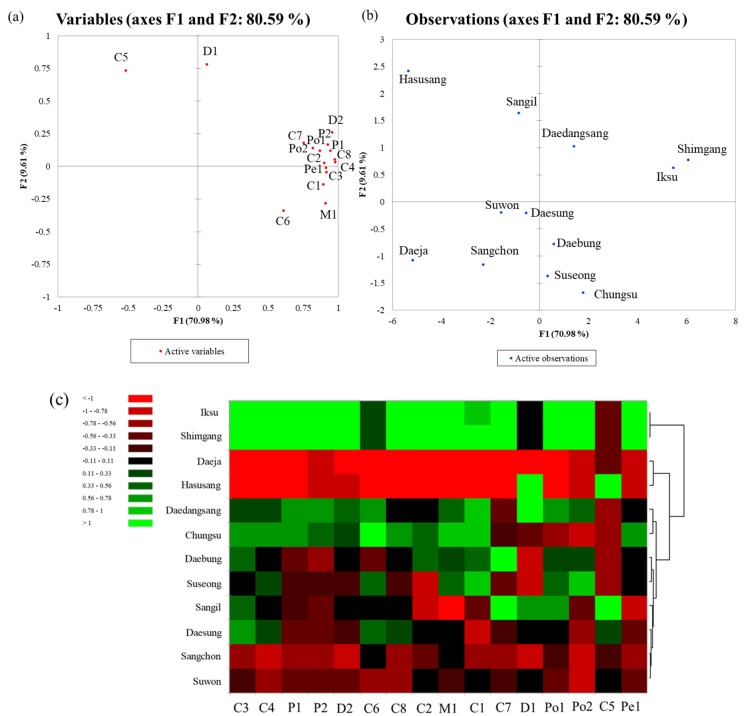
Principal component analysis (PCA) loading plot (**A**), PCA score plot (**B**), and heatmap (**C**) based on anthocyanin peak areas analyzed by UHPLC-(ESI)-qTOF.

Figure 2 shows the results of principal component analysis and the heatmap based on anthocyanin compositions in 12 mulberry fruit cultivars. The first two principal components, F1 and F2, explained 80.59% of the total variables, accounting for 70.98% and 9.61%, respectively. Shimgang and Iksu were separated from the other cultivars and were highly correlated with most anthocyanins. Interestingly, Hasusang mulberry was correlated with cyanidin malonyl hexose hexoside.

The heatmap showed that the Shimgang and Iksu cluster contained higher anthocyanin contents than other cultivars, except for cyanidin malonyl hexose hexoside. The cluster of Daeja and Hasusang exhibited lower anthocyanin contents than other cultivars, except for delphinidin dihexoside and cyanidin malonyl hexose hexoside. Cyanidin based glycosides are responsible for red color. Mulberry cultivars (i.e., Shimgang and Iksu,) containing higher cyanidin glycosides than other cultivars may be recommended to be used as a natural colorant with high antioxidant activity in foods and other foodstuffs [37,38].

### 3.6. Pearson’s Correlation between Anthocyanin Compounds and Antioxidant Activities

Table 5 displays Pearson correlations among anthocyanin contents, TPC, TFC, and antioxidant activities. To simplify data presentation, the sixteen identified anthocyanins were grouped into six categories (cyanidin, delphinidin, pelargonidin, peonidin, malvidin, and petunidin) by combining conjugates. TFC and TPC were significantly correlated with most anthocyanin levels in mulberry fruits (*p* < 0.05). Antioxidant activities (DPPH radical scavenging activity and FRAP results) were significantly correlated with most anthocyanin contents, except for delphinidin dihexoside and cyanidin malonyl hexose hexoside contents (*p* < 0.05). Degree of hydroxylation and methoxylation in the B ring of anthocyanins affect antioxidant activity. Additionally, glycosylation pattern affects antioxidant activity of anthocyanins. The matrix also affects antioxidant potential. A previous study showed that the aglycones show generally higher antioxidant activities than the glycosides of anthocyanins in LDL; however, glycosides have higher antioxidant power compared to aglycones in bulk oils [39].

### 3.7. Antioxidant Activity, TPC, TFC, and Anthocyanin Composition of Mulberry Syrup

Table 6 shows the antioxidant activities, TPC, TFC, and anthocyanin compositions of mulberry syrups with various citric acid levels (0%−0.3%). The results were also determined for solid residues, a byproduct of mulberry syrup production. Mulberry fruits showed higher antioxidant activities than solid residues regardless of acid levels (*p* < 0.05).

The antioxidant activity, TPC, and TFC of solid residues and syrup increased with increasing citric acid levels (*p* < 0.05). Cyanidin-3-*O*-glucoside, cyanidin-3-*O*-rutinoside, and pelargonidin-3-*O*-glucoside concentrations in solid residues were higher with citric acid addition compared to the control (0% citric acid). Anthocyanin stability is influenced by pH value [40]. In a previous study, the anthocyanin degradation rates of black carrots, red cabbages, and grape skin were higher at pH 3 (2.6%–11.8%) than at pH 7 (15.0%–49.8%) [41]. Therefore, the addition of citric acid may lower the pH, contributing to improved anthocyanin stability in the solid residues. Hubbermann et al. reported that the red color stability (measured by a^*^ value) of elderberry concentrate was improved after 1 month of storage when citric acid (0.2 M, pH 3.9) was added, compared with ascorbic acid [40]. Thus, the type organic acids added may affect the stability of anthocyanins contained in berry syrup, which in turn affects the stability of the color of the syrup. Interestingly, in the syrup, the anthocyanin concentrations were not affected by adding citric acid; however, antioxidant activity, TPC, and TFC increased with increasing acid levels. Thus, the products of anthocyanin degradation in the syrup with citric acid may be retained well at low pH and may have contributed to high antioxidant activity, TPC, and TFC.

Syrup showed lower antioxidant activity compared to solid residues (*p* < 0.05); this may be explained by higher TPC, TFC, and anthocyanin levels in solid residues compared to the syrup. Thus, the consumption of mulberry syrup with solid residues is recommended. A previous study showed that anthocyanin degradation rate is faster in syrups with lower anthocyanin contents in strawberry and blackcurrant syrups [42]. Because mulberry is a rich source of anthocyanins compared to other berries, mulberry syrup can be applied to various food stuffs such as beverage, jelly, bakery products. When citric acid and sucrose were added to soft drinks together, color stability (determined by a^*^ value) was improved more than that of citric acid alone after 35 days of storage [40]. The mulberry syrup is commonly added to carbonated drink. Thus, it is recommended to add citric acid to make mulberry syrup. A previous study reported that encapsulation improved anthocyanin stability in the anthocyanin fortified jelly [43]. To stabilize anthocynin pigments, encapsulation technology (e.g., spray-drying with arabic and maltodextrin) can be applied before fortification of the mulberry extract.

## 4. Conclusions

The antioxidant activity, TPC, TFC, anthocyanin compositions of 12 mulberry fruit cultivars were investigated. Sixteen anthocyanins (8 cyanidin derivatives, 2 delphinidin derivatives, 2 pelargonidin derivatives, 1 malvidin derivative, 1 petunidin derivative, and 2 peonidin derivatives) were identified using UHPLC-(ESI)-qTOF. Among them, six anthocyanins (i.e., malvidin hexoside, cyanidin malonyl hexose hexoside, cyanidin pentoside, cyanidin malonyl hexoside, petunidindeoxyhexose hexoside, and cyanidin deoxyhexoside) were tentatively identified for the first time in mulberry fruits. The major anthocyanins of mulberry fruits were cyanidin-3-*O*-glucoside and cyanidin-3-*O*-rutinoside, which were highest in Iksu compared to other cultivars. The high anthocyanin contents of Iksu mulberries may explain its high antioxidant activity, TPC, and TFC. Furthermore, when acid was added during syrup production, antioxidant activity was improved. Additionally, solid residues, a byproduct of syrup, contained high anthocyanin levels; thus, it is recommended to consume solid residues with mulberry syrup. Also, the freeze-dried mulberry extract and/or syrup can be applied to foodstuffs such as beverages, jelly, bakery products, and cosmetics etc.

## Figures and Tables

**Table 1:** Mulberry sample information.

Cultivar	Breeding Year	Genetic Information	Species	Fruit Weight (g)	Soluble Solid Content (°Brix)
Daedangsang	unknown	unknown	*Morus Lhou(Ser.)* Koidz.	2.57 ± 0.23	10.5 ± 0.1
Daebung	2007	Daedosang 4X	*Morus Lhou(Ser.)* Koidz.	2.64 ± 0.18	13.8 ± 0.0
Daeja	2007	Kuksang No. 20 4X	*Morus Lhou(Ser.)* Koidz.	2.46 ± 0.23	15.0 ± 0.1
Daesung	2005	Ficux 4X	*Morus Lhou(Ser.)* Koidz.	3.20 ± 0.33	11.4 ± 0.1
Sangchon	2011	unknown	*Morus Alba* L.	2.59 ± 0.20	17.2 ± 0.0
Suwon	1983	unknown	*Morus Alba* L.	2.67 ± 0.14	18.3 ± 0.1
Hasusang	1883	unknown	*Morus Alba* L.	1.70 ±0.29	13.8 ± 0.0
Iksu	unknown	unknown	*Morus Alba* L.	2.82 ± 0.23	12.3 ± 0.1
Suseong	1989	Jamsang No.101	*Morus Alba* L.	2.45 ± 0.21	12.5 ± 0.1
Sangil	1992	Chungil x Kuksang No.21	*Morus Alba* L.	1.82 ± 0.22	12.5 ± 0.1
Shimgang	2015	unknown	*Morus Microphylla* Buckl.	2.71 ± 0.16	12.4 ± 0.0
Chungsu	unknown	unknown	*Morus* spp.	2.63 ± 0.23	12.5 ± 0.0

**Table 2:** Total phenolic content, total flavonoid content, and antioxidant activities of 12 mulberry cultivars.

Sample	DPPH	FRAP	TPC	TFC
Daedangsang	24.40 ± 1.01 ^e^	48.89 ± 6.87 ^cd^	24.92 ± 1.18 ^b^	2.45 ± 0.50 ^bc^
Daebung	27.14 ± 0.81 ^d^	47.33 ± 4.15 ^cd^	24.62 ± 2.83 ^bc^	2.32 ± 0.40 ^bcd^
Daeja	5.85 ± 0.77 ^j^	1.33 ± 0.12 ^g^	5.68 ± 0.20 ^g^	0.65 ± 0.06 ^f^
Daesung	29.11 ± 1.65 ^cd^	50.11 ± 6.31 ^c^	26.40 ± 1.07 ^b^	2.34 ± 0.61 ^bcd^
Sangchon	13.47 ± 0.94 ^h^	26.05 ± 2.81 ^e^	19.86 ± 0.91 ^de^	1.38 ± 0.28 ^ef^
Suwon	17.91 ± 1.26 ^g^	40.76 ± 2.42 ^cd^	19.52 ± 0.79 ^e^	1.64 ± 0.31 ^cde^
Hasusang	9.57 ± 1.03 ^i^	10.60 ± 1.42 ^f^	9.46 ± 0.17 ^f^	1.54 ± 0.34 ^de^
Iksu	37.45 ± 2.09 ^b^	82.87 ± 6.69 ^a^	40.46 ± 1.81 ^a^	3.49 ± 0.53 ^a^
Suseong	22.65 ± 2.38 ^e^	39.98 ± 5.06 ^d^	22.01 ± 0.69 ^cde^	2.19 ± 0.42 ^bcd^
Sangil	20.25 ± 1.25 ^f^	44.28 ± 5.39 ^cd^	22.28 ± 0.92 ^cd^	2.52 ± 0.40 ^b^
Shimgang	40.73 ± 1.06 ^a^	73.03 ± 7.08 ^b^	38.31 ± 3.08 ^a^	3.70 ± 0.59 ^a^
Chungsu	31.21 ± 0.80 ^c^	49.28 ± 4.98 ^cd^	26.59 ± 0.69 ^b^	2.29 ± 0.44 ^bcd^

DPPH, 2,2-diphenyl-1-picrylhydrazyl; FRAP, ferric ion reducing antioxidant power; TPC, total phenolic content; TFC, total flavonoid content. DDPH and FRAP are measured as mg trolox equivalent/g dry weight (DW), TPC as mg GAE/g DW, and TFC as mg quercetin equivalent/g DW. Values are mean ± standard deviation. Mean values followed by different letters within a column are significantly different (*p* < 0.05).

**Table 3:** Anthocyanin contents (mg/g DW) in 12 mulberries cultivar determined by high-performance liquid chromatography.

Cultivars	Cyanidin-3-*O*-Glucoside	Cyanidin-3-*O*-Rutinoside	Pelargonidin-3-*O*-Glucoside	Sum
Daedangsang	10.08 ± 0.46 ^ef^	4.81 ± 0.20 ^c^	0.26 ± 0.00 ^b^	15.16 ± 0.65 ^d^
Daebung	11.87 ± 0.03 ^e^	4.44 ± 0.00 ^d^	0.05 ± 0.00 ^e^	16.36 ± 0.03 ^c^
Daeja	0.33 ± 0.00 ^j^	0.18 ± 0.00 ^i^	n.d.	0.51 ± 0.00 ^h^
Daesung	12.03 ± 0.22 ^de^	4.69 ± 0.08 ^c^	0.09 ± 0.00 ^d^	16.81 ± 0.30 ^c^
Sangchon	5.35 ± 0.11 ^i^	2.15 ± 0.03 ^h^	0.04 ± 0.02 ^e^	7.54 ± 0.16 ^g^
Suwon	8.11 ± 0.18 ^h^	2.95 ± 0.06 ^g^	0.11 ± 0.00 ^cd^	11.17 ± 0.24 ^f^
Hasusang	0.62 ± 0.01 ^j^	0.33 ± 0.00 ^i^	n.d.	0.95 ± 0.01 ^h^
Iksu	19.51 ± 0.00 ^a^	8.58 ± 0.00 ^a^	0.51 ± 0.00 ^a^	28.61 ± 0.00 ^a^
Suseong	8.91 ± 0.10 ^g^	4.37 ± 0.03 ^d^	0.11 ± 0.01 ^c^	13.40 ± 0.12 ^e^
Sangil	9.40 ± 0.05 ^g^	3.65 ± 0.02 ^f^	0.08 ± 0.03 ^d^	13.13 ± 0.03 ^e^
Shimgang	18.92 ± 0.38 ^b^	8.65 ± 0.16 ^a^	0.48 ± 0.01 ^a^	28.05 ± 0.55 ^a^
Chungsu	12.57 ± 0.11 ^c^	5.08 ± 0.04 ^b^	0.27 ± 0.01 ^b^	17.92 ± 0.16 ^b^

Values are mean ± standard deviation. Mean values followed by different letters within a column are significantly different (*p* < 0.05).

**Table 4:** Identified anthocyanins in mulberry fruits using UHPLC-(ESI)-qTOF.

Peak no.	Code	RT^a^ (min)	Compound Assigned	Molecular Formula	Predicted MS1 *m*/*z*	Fragment *m*/*z*	Error (ppm)
1	C1	4.2	Cyanidin hexose deoxyhexose hexoside	C_33_H_41_O_20_	757.2186	595 [M – h (–162) + H] ^+^ 449 [M – h (–162) – dh (–146) + H] ^+^ 287 [M – h (–162) – dh (–146) – h (–162) + H] ^+^	−1.95
2	C2	5.7	Cyanidin dihexoside	C_27_H_31_O_16_	611.1607	449 [M – h (–162) + H] ^+^ 287 [M – h (–162) – h (–162) + H] ^+^	−0.01
3	D1	5.8	Delphinidin dihexoside	C_27_H_31_O_17_	627.1556	465 [M – h (–162) + H] ^+^ 303 [M – h (–162) – h (–162) + H] ^+^	−0.09
4	C3	6.4	Cyanidin 3-*O*-glucoside^*^	C_21_H_21_O_11_	449.1078	287 [M – h (–162) + H] ^+^	−0.22
5	C4	7.2	Cyanidin 3-*O*-rutinoside^*^	C_27_H_31_O_15_	595.1657	449 [M – dh (–146) + H] ^+^ 287 [M – dh (–146) – h (–162) + H] ^+^	−1.99
6	P1	7.8	Pelargonidin 3-*O*-glucoside^*^	C_21_H_21_O_10_	433.1129	271 [M – h (–162) + H] ^+^	0.27
7	M1	8.6	Malvidin hexoside	C_23_H_25_O_12_	493.1341	331 [M – h (–162) + H] ^+^	0.28
8	P2	8.6	Pelargonidin deoxyhexose hexoside	C_27_H_31_O_14_	579.1708	433 [M – dh (–146) + H] ^+^ 271 [M – dh (–146) – h (–162) + H] ^+^	−0.04
9	Po1	8.8	Peonidin hexoside	C_22_H_23_O_11_	463.1235	301 [M – h (–162) + H] ^+^	−0.68
10	C5	8.9	Cyanidin malonyl hexose hexoside	C_30_H_33_O_19_	697.1611	535 [M – h (–162) + H] ^+^ 449 [M – h (–162) – mal (–86) + H] ^+^ 287 [M – h (–162) – mal (–86) – h (–162) + H] ^+^	−0.12
11	C6	9.3	Cyanidin pentoside	C_20_H_19_O_10_	419.0973	287 [M – p (–132) + H] ^+^	−0.22
12	Po2	9.6	Peonidin deoxyhexose hexoside	C_28_H_33_O_15_	609.1814	463 [M – dh (–146) + H] ^+^ 301 [M – dh (–146) – h (–162) + H] ^+^	0.05
13	C7	10.6	Cyanidin malonyl hexoside	C_24_H_23_O_14_	535.1082	287 [M – h (–162) – mal (–86) + H] ^+^	0.56
14	Pe1	10.7	Petunidin deoxyhexose hexoside	C_28_H_33_O_16_	625.1763	479 [M – dh (–146) + H] ^+^ 317 [M – dh (–146) – h (–162) + H] ^+^	−1.23
15	C8	11.4	Cyanidin deoxyhexoside	C_21_H_21_O_10_	433.1129	287 [M – dh (–146) + H] ^+^	−0.59
16	D2	11.5	Delphinidin deoxyhexose hexoside	C_27_H_31_O_16_	611.1607	465 [M – dh (–146) + H] ^+^ 303 [M – dh (–146) – h (–162) + H] ^+^	0.30

UHPLC-(ESI)-qTOF, ultra-high-performance liquid chromatography coupled with electrospray ionization/quadrupole time-of-flight; RT^a^, retention time of the peak; h, hexose; dh, deoxyhexose; p, pentose; mal, malonic acid moiety. * identified with authentic standard. +*m*/*z* are based on protonated pseudomolecular ions ([M + H]^+^).

**Table 5:** Pearson correlations among anthocyanin compounds, total phenolic and flavonoid contents, and antioxidant activities (DPPH and FRAP).

	Cyanidin	Delphinidin	Pelargonidin	Peonidin	Malvidin	Petunidin	TPC	TFC	DPPH	FRAP	L	a
b	−0.279	−0.475	−0.487	−0.34	−0.214	−0.234	−0.357	−0.41	−0.309	−0.397	−0.911^**^	0.771^**^
a	−0.197	−0.296	−0.361	−0.335	−0.273	−0.139	−0.217	−0.229	−0.163	−0.249	−0.955^**^	
L	0.188	0.345	0.392	0.289	0.203	0.15	0.229	0.276	0.188	0.274		
FRAP	0.970^**^	0.937^**^	0.884^**^	0.876^**^	0.866^**^	0.809^**^	0.985^**^	0.945^**^	0.956^**^			
DPPH	0.968^**^	0.915^**^	0.872^**^	0.810^**^	0.879^**^	0.875^**^	0.960^**^	0.934^**^				
TFC	0.927^**^	0.971^**^	0.877^**^	0.892^**^	0.756^**^	0.827^**^	0.941^**^					
TPC	0.965^**^	0.940^**^	0.899^**^	0.880^**^	0.893^**^	0.846^**^						
Petunidin	0.784^**^	0.894^**^	0.928^**^	0.746^**^	0.826^**^							
Malvidin	0.872^**^	0.772^**^	0.828^**^	0.756^**^								
Peonidin	0.854^**^	0.882^**^	0.802^**^									
Pelargonidin	0.836^**^	0.954^**^										
Delphinidin	0.889^**^											

DPPH, 2,2-diphenyl-1-picrylhydrazyl; FRAP, ferric ion reducing antioxidant power; TPC, total phenolic content; TFC, total flavonoid content; L, whiteness; a, redness; b, yellowness. ** significantly correlated (*p* < 0.01). Conjugates of cyanidin, delphinidin, pelargonidin, peonidin, malvidin, and petunidin were summed to investigate Pearson correlations.

**Table 6:** Antioxidant activity (DPPH), total phenolic contents, total flavonoid contents, and anthocyanin contents of mulberry fruits, solid residues, and syrup (µg/g FW).

		DPPH	TPC	TFC	Cyanidin-3-*O*-glucoside	Cyanidin-3-*O*-rutinoside	Pelargonidin-3-*O*-glucoside	Sum of Anthocyanins
Mulberry fruit		3.26 ± 0.26 ^A^	3.49 ± 0.20 ^A^	0.38 ± 0.04 ^A^	1312.04 ± 62.82 ^A^	775.17 ± 34.27 ^A^	24.83 ± 0.32 ^A^	2112.04 ± 96.77 ^A^
Solid residues	Citric acid 0%	1.10 ± 0.04 ^D^	1.25 ± 0.06 ^D^	0.20 ± 0.03 ^C^	213.65 ± 6.80 ^C^	98.59 ± 2.11 ^C^	n.d.	312.25 ± 8.90 ^C^
	Citric acid 0.2%	1.30 ± 0.07 ^C^	1.42 ± 0.06 ^C^	0.24 ± 0.04 ^C^	361.69 ± 20.53 ^B^	170.89 ± 16.35 ^B^	7.00 ± 0.36 ^B^	539.27 ± 37.24 ^B^
	Citric acid 0.3%	1.59 ± 0.04 ^B^	1.64 ± 0.12 ^B^	0.31 ± 0.04 ^B^	410.97 ± 20.46 ^B^	186.50 ± 9.13 ^B^	7.32 ± 0.38 ^B^	604.79 ± 29.97 ^B^
Syrup	Citric acid 0%	0.43 ± 0.03 ^b^	0.42 ± 0.03 ^b^	0.08 ± 0.02 ^c^	81.23 ± 5.57 ^a^	72.99 ± 3.24 ^a^	n.d.	147.23 ± 2.81 ^a^
	Citric acid 0.2%	0.47 ± 0.02 ^b^	0.47 ± 0.03 ^ab^	0.11 ± 0.01 ^b^	84.81 ± 7.98 ^a^	74.81 ± 2.16 ^a^	n.d.	162.31 ± 4.22 ^a^
	Citric acid 0.3%	0.78 ± 0.16 ^a^	0.57 ± 0.06 ^a^	0.15 ± 0.02 ^a^	104.80 ± 10.83 ^a^	77.02 ± 2.68 ^a^	n.d.	171.71 ± 5.54 ^a^

DPPH, 2,2-diphenyl-1-picrylhydrazyl; TPC, total phenolic content; TFC, total flavonoid content. DDPH is measured as mg trolox equivalent/g fresh weight (FW), TPC as mg GAE/g FW, and TFC as mg quercetin equivalent/g FW. Values are mean ± standard deviation. Mean values followed by different capital letters indicate significant differences for mulberry fruit and solid residue of mulberry syrup at *p* < 0.05. Mean values followed by different lower case letters indicate significant differences for mulberry syrup at *p* < 0.05.

## Supplementary Materials

The following are available online at https://www.mdpi.com/2076-3921/9/3/242/s1, Figure S1: A representative UHPLC-(ESI)-qTOF extracted ion chromatogram of anthocyanins of mulberry fruits (cv. Shimgang); Table S1: Hunter color values of 12 mulberry fruit cultivars; Table S2: Average peak areas of anthocyanins in 12 mulberry cultivars determined by UHPLC-(ESI)-qTOF.
