# Supplementary material for: Variations in Anthocyanin Profiles and Antioxidant Activity of 12 Genotypes of Mulberry (Morus spp.) Fruits and Their Changes during Processing
Source: Antioxidants (Basel). 2020 Mar 17;9(3):242. doi: 10.3390/antiox9030242 (PMC7139643; doi:10.3390/antiox9030242)
Supplement: Supplementary file 1 [file antioxidants-09-00242-s001.pdf]

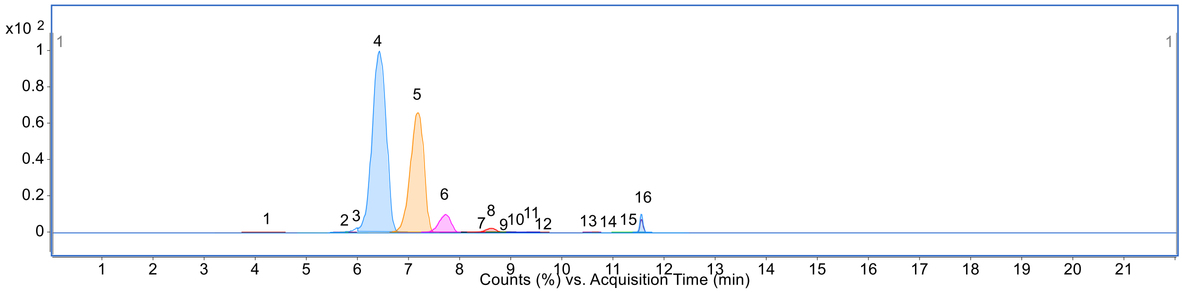

Figure S1. A representative UHPLC-(ESI)-qTOF extracted ion chromatogram of anthocyanins of mulberry fruits (cv. Shimgang)

Table S1. Hunter color values of 12 mulberry fruit cultivars.

**Cultivar**	**L**	**a**	**b**
Daedangsang	45.21 ± 0.04 d	72.29 ± 0.07 e	58.07 ± 0.29 g
Daebung	43.38 ± 0.07 c	71.83 ± 0.12 c	62.01 ± 1.00 i
Daeja	80.99 ± 0.02 k	29.75 ± 0.11 a	-1.16 ± 0.08 a
Daesung	46.21 ± 0.05 f	72.84 ± 0.03 f	52.67 ± 0.84 f
Sangchon	49.63 ± 0.04 i	73.10 ± 0.13 g	33.61 ± 0.33 c
Suwon	49.53 ± 0.05 h	73.92 ± 0.17 h	40.38 ± 0.45 d
Hasusang	59.80 ± 0.05 j	60.93 ± 0.12 b	5.54 ± 0.12 b
Iksu	41.45 ± 0.03 a	71.81 ± 0.09 c	68.33 ± 0.41 j
Suseong	46.23 ± 0.03 f	72.69 ± 0.10 f	50.46 ± 0.67 e
Sangil	46.48 ± 0.02 g	72.04 ± 0.12 d	50.24 ± 0.75 e
Shimgang	41.68 ± 0.03 b	72.19 ± 0.12 e	68.82 ± 0.41 j
Chungsu	45.88 ± 0.03 e	72.72 ± 0.13 f	59.22 ± 0.65 h

L, whiteness; a, redness; b, yellowness. Data are presented as mean ± standard deviation. Values followed by different letters for different mulberry cultivars are significantly different by Duncan's multiple range test ($p < 0.05$).

Table S2. Average peak areas of anthocyanins in 12 mulberry cultivars determined by UHPLC-(ESI)-qTOF

Code	Daedangsang	Daebung	Daeja	Daesung	Sangchon	Suwon	Hasusang	Iksu	Suseong	Sangil	Shimgang	Chungsu
C1	112,961 b	97,949 c	n.d.	24,540 g	35,285 f	69,503 d	n.d.	118,701 b	114,515 b	54,484 e	140,797 a	114,957 b
C2	153,277 d	217,730 c	n.d.	147,865 d	104,362 e	154,491 d	12,868 g	346,805 b	37,943 f	54,248 f	384,431 a	204,372 c
D1	114,854 a	29,027 g	19,695 h	50,592 de	29,338 g	53,956 d	81,901 b	49,787 e	27,478 g	69,485 c	51,256 de	37,616 f
C3	128,421,160 d	140,840,112 c	10,370,079 h	151,327,227 b	85,260,003 f	101,488,582 e	17,833,986 g	185,083,015 a	123,542,953 d	142,511,805 c	185,113,101 a	156,752,286 b
C4	63,008,260 de	56,351,466 fg	2,418,047 k	64,020,842 d	26,827,882 i	33,002,149 h	6,496,230 j	101,209,401 b	59,309,324 ef	53,999,239 g	105,924,221 a	72,093,394 c
P1	9,705,565 b	3,628,431 d	835,955 f	4,265,933 c	2,654,348 e	4,289,875 c	388,190 f	16,409,907 a	4,911,138 c	4,722,313 c	15,927,082 a	10,052,244 b
M1	222,722 c	193,811 d	n.d.	178,501 de	173,718 de	164,456 e	4,661 g	321,601 a	229,003 c	67,698 f	297,284 b	282,964 b
P2	1,993,680 b	526,097 f	89,986 h	691,424 e	361,324 g	579,784 ef	84,640 h	3,484,754 a	1,031,860 d	688,109 e	3,528,788 a	1,678,495 c
Po1	58,824 b	48,566 c	n.d.	42,153 d	37,618 e	32,289 f	n.d.	73,761 a	52,251 c	57,045 b	71,749 a	27,215 g
C5	n.d.	n.d.	1,653 fg	9,626 c	4,996 e	6,857 d	42,233 a	2,104 f	n.d.	24,191 b	2,267 f	n.d.
C6	415,894 b	216,222 f	n.d.	363,026 c	265,840 e	178,357 g	30,526 h	315,981 d	374,137 c	264,096 e	303,632 d	659,821 a
Po2	18,019 d	17,802 d	n.d.	3,429 f	n.d.	n.d.	n.d.	37,552 b	28,337 c	6,271 e	41,879 a	n.d.
C7	22,977 d	73,632 ab	n.d.	28,031 c	15,517 e	31,435 c	n.d.	71,797 b	22,823 d	71,090 b	75,922 a	31,162 c
Pe1	9,141 d	8,140 de	n.d.	4,694 ef	1,710 fg	5,036 def	n.d.	21,706 b	8,955 d	n.d.	31,589 a	15,576 c
C8	194,595 d	196,110 d	n.d.	221,537 c	93,198 f	99,587 f	14,788 g	403,138 a	169,998 e	184,180 de	416,017 a	271,168 b
D2	2,321,760 c	1,850,339 e	266,704 j	1,682,548 f	857,630 i	1,251,736 h	786,544 i	3,963,248 b	1,530,070 g	1,972,706 de	4,128,740 a	2,030,557 d
SUM	206,773,689 d	204,295,434 d	14,002,119 i	223,061,968 c	116,722,769 g	141,408,093 f	25,776,567 h	311,913,258 a	191,390,785 e	204,746,960 d	316,438,755 a	244,251,827 b

n.d., not detected. Compound codes are shown in Table 4.
